# GC-TOF/MS-based metabolomics analysis to investigate the changes driven by N-Acetylcysteine in the plant-pathogen *Xanthomonas citri* subsp. *citri*

**DOI:** 10.1038/s41598-021-95113-4

**Published:** 2021-07-30

**Authors:** Simone Cristina Picchi, Mariana de Souza e Silva, Luiz Leonardo Saldanha, Henrique Ferreira, Marco Aurélio Takita, Camila Caldana, Alessandra Alves de Souza

**Affiliations:** 1 Centro de Citricultura “Sylvio Moreira” – Instituto Agronômico de Campinas, Cordeirópolis, São Paulo, 13490-970 Brazil; 2 Departamento de Bioquímica e Microbiologia, Instituto de Biociências, Universidade Estadual Paulista, Rio Claro, São Paulo 13506-900 Brazil; 3 Laboratório Nacional de Ciência e Tecnologia do Bioetanol - Centro Nacional de Pesquisa em Energia e Materiais, Campinas, São Paulo, 13083-100 Brazil; 4 Present Address: Max-Planck-Institut Für Molekulare Pflanzenphysiologie, Wissenschaftspark Golm, Am Mühlenberg 1, 14476 Potsdam, Germany

**Keywords:** Antimicrobials, Pathogens

## Abstract

N-Acetylcysteine (NAC) is an antioxidant, anti-adhesive, and antimicrobial compound. Even though there is much information regarding the role of NAC as an antioxidant and anti-adhesive agent, little is known about its antimicrobial activity. In order to assess its mode of action in bacterial cells, we investigated the metabolic responses triggered by NAC at neutral pH. As a model organism, we chose the Gram-negative plant pathogen *Xanthomonas citri* subsp. *citri* (*X. citri*), the causal agent of citrus canker disease, due to the potential use of NAC as a sustainable molecule against phytopathogens dissemination in citrus cultivated areas. In presence of NAC, cell proliferation was affected after 4 h, but damages to the cell membrane were observed only after 24 h. Targeted metabolite profiling analysis using GC–MS/TOF unravelled that NAC seems to be metabolized by the cells affecting cysteine metabolism. Intriguingly, glutamine, a marker for nitrogen status, was not detected among the cells treated with NAC. The absence of glutamine was followed by a decrease in the levels of the majority of the proteinogenic amino acids, suggesting that the reduced availability of amino acids affect protein synthesis and consequently cell proliferation.

## Introduction

Bacterial biofilms are responsible for many diseases in plants, animals and humans in which biofilm formation is an essential step for host colonization and disease development^1–4^. The difficulty for biofilm eradication in clinical and environmental settings resides in their multiple resistance mechanisms such as poor antimicrobial penetration, slow growth, adaptive stress responses and formation of persister cells^5–9^. You cannot alter accepted Supplementary Information files except for critical changes to scientific content. If you do resupply any files, please also provide a brief (but complete) list of changes. If these are not considered scientific changes, any altered Supplementary files will not be used, only the originally accepted version will be published.We do not have alterations to make.

The bacterium *Xanthomonas citri* subsp. *citri,* the causing agent of citrus canker disease, is one of the most destructive phytopathogen in the citrus agribusiness^10^. Among its virulence factors, biofilm formation plays an essential role at early stages of infection by enhancing bacterial epiphytic survival^11–14^. Importantly, multiple mutants of *X. citri* impaired in biofilm formation consistently exhibit a decrease in bacterial growth *in planta* and have reduced ability to elicit canker symptoms in susceptible host^15^. This led us to hypothesize that compounds inhibiting biofilm formation may reduce its infection and enhance the control of citrus canker disease. Indeed, it was previously verified that N-Acetylcysteine (NAC), a cysteine analogue known to disrupt disulphide bonds in bacterial biofilm^16^, was able to disrupt biofilm formation in *X. citri* as well as kill bacterial cells leading to a decrease in plant disease^17^.

NAC is largely used in humans to decrease biofilm-based infections due to its properties as a mucolytic agent that breaks biofilms and also improves body healthy as an antioxidant molecule^16, 18–20^. Furthermore, NAC has antimicrobial effect inhibiting growth of many different Gram-negative and Gram-positive bacteria^8, 20–24^. Therefore, NAC is emerging as an interesting potential therapeutics molecule since it does not induce genetic resistance and is beneficial to human health^9, 16^.

Despite the well-established role of NAC as a mucolytic agent and an antioxidant molecule, the mode of action of this compound in triggering death of prokaryotic cells is still unknown. It has been shown that due to the acid trait of NAC (pH < pKa) it can penetrates in the biofilm matrix and eventually kill 100% of the bacteria embedded in the biofilm^9^, however many authors have shown the antimicrobial effect of NAC even at neutral pH^8, 17, 20, 25, 26^. These results indicate that other factors might be also involved with the NAC antimicrobial property.

In this study we used the Gram-negative plant-pathogen *X. citri* as model to investigate the impact of NAC treatment in the primary metabolism of prokaryotic cells. By performing a time course analysis, we found out that growth started to be impaired after 4 h of NAC treatment, matching the metabolic alterations in most of the proteogenic amino acids. Our findings indicated that NAC interferes with nitrogen metabolism, reducing the availability of amino acids for protein synthesis, which might contribute to reduction in cell proliferation and activation of cell death. A molecular network was created, and compounds annotation was made on the basis of comparison with spectral database. When the metabolomics multivariate data analysis (MVDA) results were integrated in a multi-informational molecular network (MN)^27^, this approach highlighted metabolites that differed significantly with NAC treatment.

## Results

### Effect of NAC on bacterial cell growth

In previous experiments, we verified that 8 mg/mL of NAC was able to kill *X. citri* cells after 24 h of growth in a population starting from 10^4^ colony forming unit (CFU)/mL at the beginning of the treatment^17^. In this study, we analyzed the *X. citri* growth curve in the presence of 8 mg/mL of NAC for 24 h to understand the primary metabolic changes that affect cell growth. In order to assess the metabolic changes, we used an initial population of 10^6^ CFU/mL instead of 10^4^ CFU/mL used by Picchi et al.^17^. With the higher cell population NAC impairs growth without killing all the cells, making it possible to evaluate the NAC effect on the cell metabolism (Supplementary Fig. S1).

**Figure 1:**
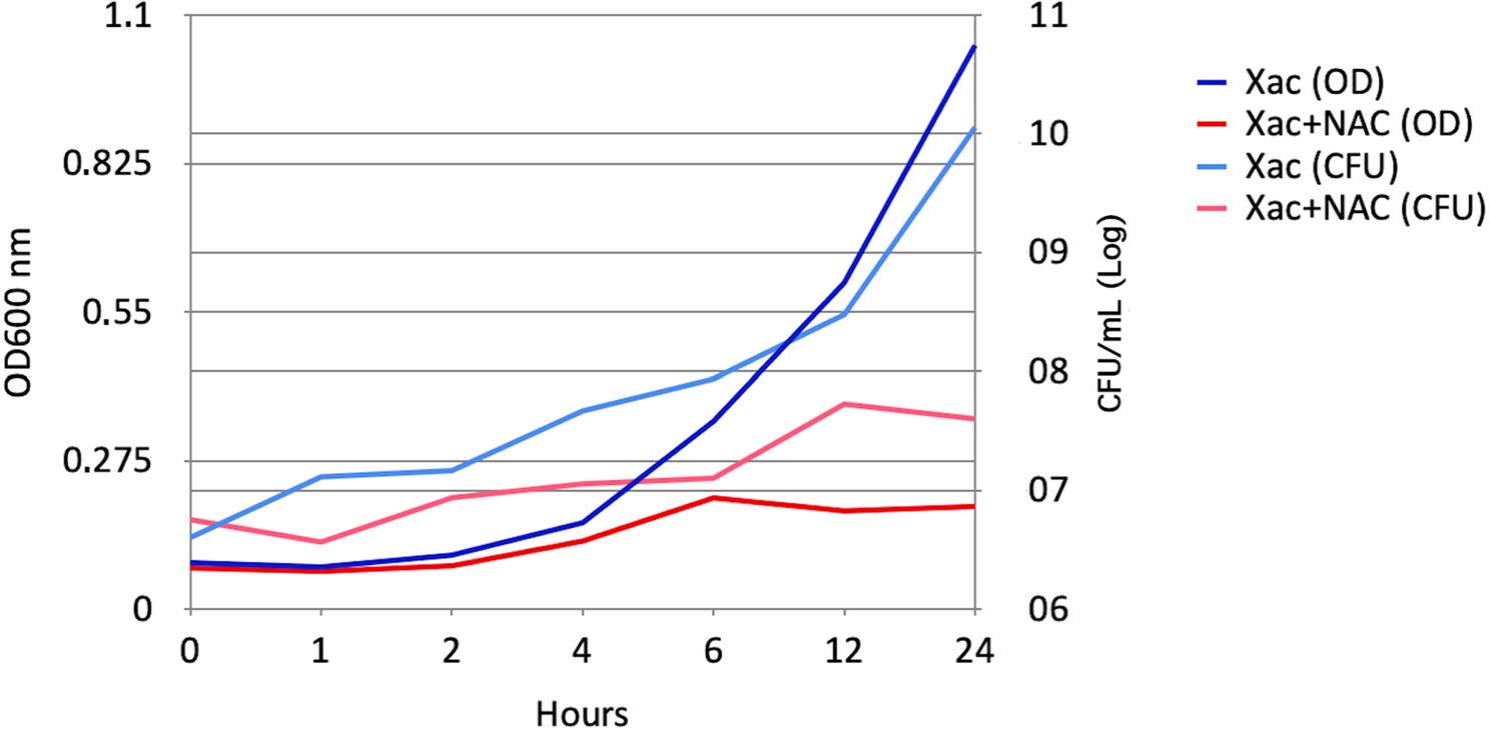
Growth curve profiles of *Xanthomonas citri* subsp. *citri* in presence of NAC. Six time points 1, 2, 4, 6 12 and 24 h were evaluated after 8 mg/mL of NAC addition as well as non-treated control through OD and CFU measures. After 6 h, a significant lower growth was observed for the bacteria treated with NAC (*P* < 0.001).

**Figure 2:**
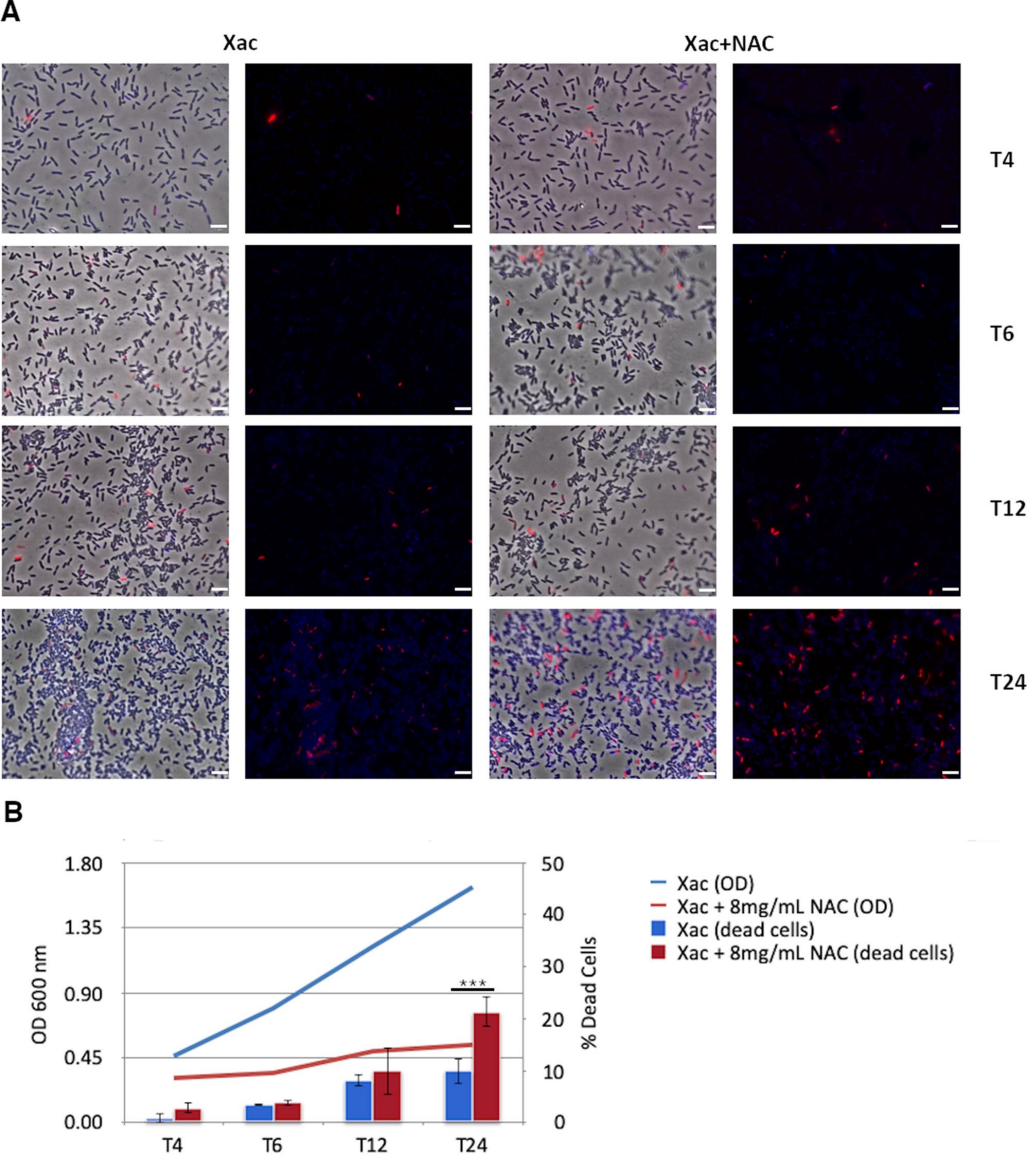
Cellular permeability of *Xanthomonas citri* subsp. *citri* in the presence of 8 mg/mL of N-Acetylcysteine (NAC). (**a**) Live/Dead staining was performed on *X. citri* cells following a 0–24 h incubation with NAC. Blue-stained cells have intact membranes, whereas red-stained cells exhibit permeabilized membranes. Magnification of × 100; the scale bars on each panel represent 5 μm. (**b**) Percentage of living or dead bacterial cells following 4–24 h exposure to 8 mg/mL of NAC. At least 1000 bacterial cells were counted under a fluorescence microscope (n > 1000). The mean of two experiments with five technical replicates is shown. Asterisks show statistically significant differences using *t*-Student (***P < 0.001). Error bars represent the standard errors of the means.

Exponential growth was observed 4 h after *X. citri* inoculation, while the presence of NAC causes a significant reduction at this time point (Fig. 1). The cells concentration increased from 10^6^ to 10^10^, but it was observed a significant reduction in cell growth in presence of NAC, increasing only one log, from 10^6^ to 10^7^, i.e., three logs difference in relation to the control. Aiming to analyze whether NAC was disrupting the cell membrane, we used SYTO9 and propidium iodide (PI) methodology to assess cell viability. SYTO9 penetrates both live and dead cells, whereas PI stains only cells with corrupted cell membrane and intercalates with the nucleic acids. Despite the significant lower number of cells 4 h after NAC treatment, the percentage of dead cells did not show a significant difference with the untreated control until 12 h (Fig. 2a and Fig. 2b). However, after 24 h a significant higher number of cells stained with PI (here represented in red) was observed following treatment with NAC, when approximately 20% of cells had their membrane permeabilized. These results demonstrated that the mechanism by which NAC acts as an antimicrobial molecule precedes disruption of the cell membrane.

### Metabolic changes in *X. citri* subsp. *citri* cells exposed to NAC

To investigate changes on primary metabolism of the bacteria incubated with NAC, we performed a well-established gas chromatography-mass spectrometry (GC–MS) method^28^. Since NAC was not included in the compound reference library^29^, we first analyzed NAC standard in GC–MS. We were able to annotate two peaks at retention index (RI) of 554,630 and 622,850 and the selective masses of 218, 260, 100, 115, 364, 173 and 184, 156, 114, 232, 274, 100, respectively. These features were included in the reference library and used for the annotation of the metabolites. For NAC quantification, we always considered the peak with the highest intensity.

**Figure 3:**
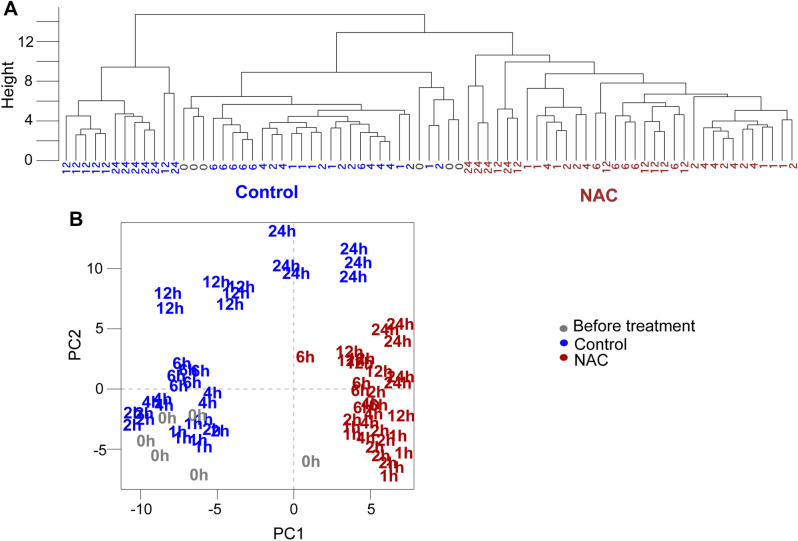
Hierarchical Clustering Analysis (**a**) and Principal Component Analysis (**b**) HCA was performed by average linkage clustering method using Euclidean distance. Samples are named according to the time points (hours) and colored coded according to treatment (before treatment -gray; control-blue and NAC-red).

As bacterial growth was already reduced after 4 h of NAC treatment, we carried out a time series experiment in which samples were harvested during the lag phase (i.e., 0, 2, 4 h) and exponential phase (6, 12 and 24 h) to better assess the influence of NAC in bacterial growth. A total of 55 metabolites with known chemical structures were identified by a targeted analysis^30^, except for glutamine all metabolites were detected in both treatments. These metabolites covered primary metabolism pathways which includes amino acid metabolism, glycolysis, and the tricarboxylic acid (TCA) cycle (Supplementary Table S1). Hierarchical clusters analyses (HCA) clearly split the samples into two main branches: one corresponding to NAC treatment and control samples (Fig. 3a). While samples treated with NAC did not show a define separation along the time points, the control samples could be further grouped into early phase (1–6 h) and late exponential (12–24 h) growth. In order to get a better overview of the metabolites leading these cluster patterns, we performed principal component analysis (Fig. 3b). Similarly, to the HCA results, in principal component analysis (PCA), PC1 separates the sample according to the treatment, explaining 46% of the variation. Not surprisingly, inspection of the loadings responsible for the PC1 separation shows NAC and cysteine largely contributed to the discrimination of the treatments followed by few amino acids and sugars (Supplementary Table S2). The dynamic of the cell proliferation was captured by PC2, which explains 26% of the variation in this dataset. This dynamic is, however, much more clearly in the control samples than in the NAC-treated samples. Such trend was mainly related to the differences in amino acids, glycerol, adenine, and trehalose. These results suggest that NAC treatment leads to metabolic rewiring which might affect cell proliferation.

### Determinants of cell growth-related metabolic changes

**Figure 4:**
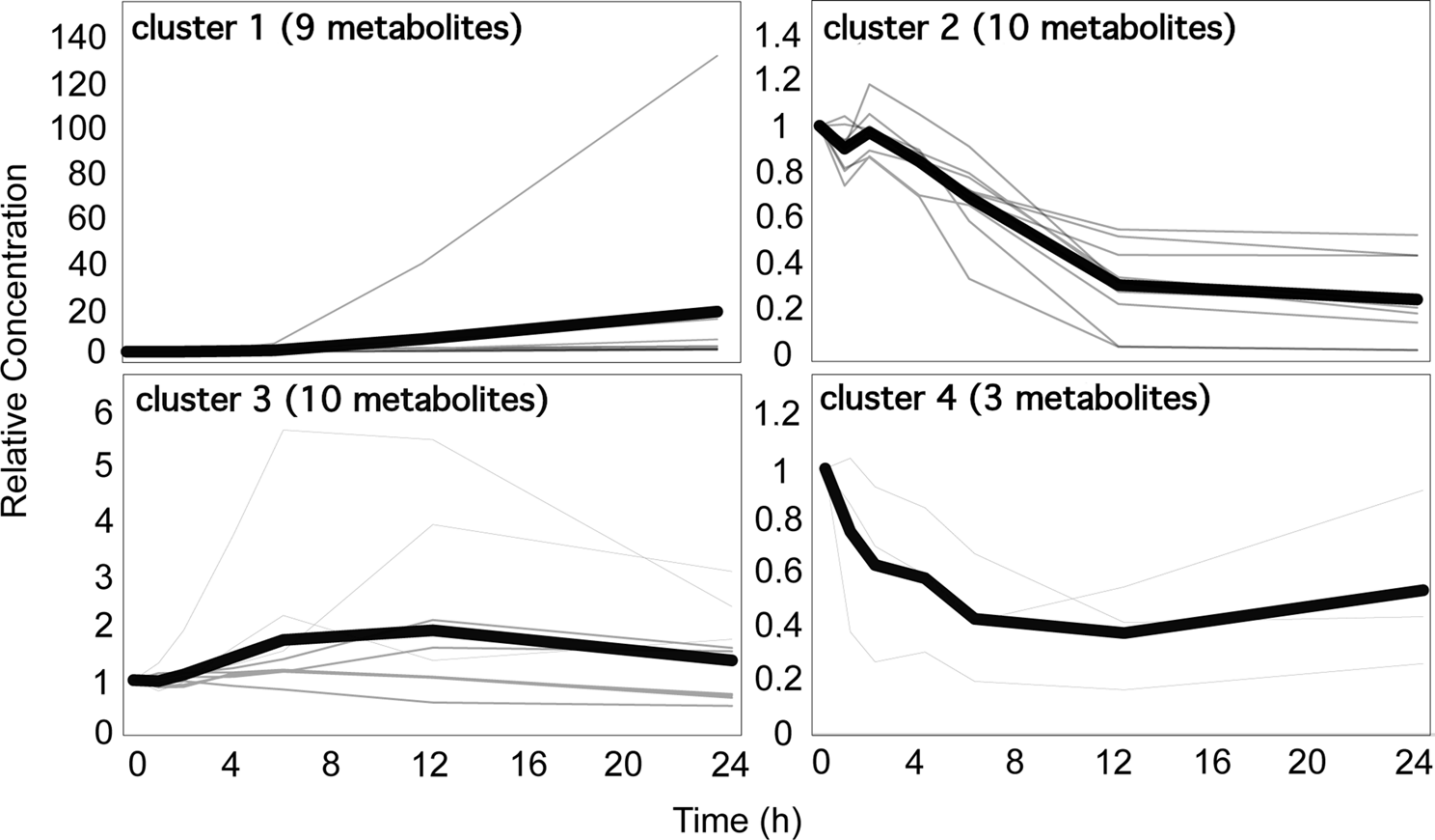
K-means clustering to infer pattern of metabolites during *X. citri* growth. Graphs represent the metabolite pattern normalized to its respective time point 0 (i.e., before treatment), which are displayed in gray. The mean of each cluster is represented by the black lines.

Metabolism directly participates in cell division and proliferation. As NAC application seems to interfere in cell proliferation, we first focused on the metabolic changes along the *X. citri* growth in the control condition. We selected only the metabolites, in which at least one time-point was significantly different with respect to the time point 0 in the control samples, resulting in 32 compounds (Table 1). In order to facilitate the comparison among the metabolites, we normalized the value of each metabolite in reference to their levels at time-point 0. We next carried out to k-means clustering analysis for the identification of metabolic modules whose patterns change along the growth curve (Fig. 4). The analysis allowed the discrimination of 4 clusters. Early changes in growth were mainly captured by the clusters 2, 3 and 4. Despite the slightly different dynamic in the response, clusters 3 and 4 includes metabolites whose levels follow a rapid lowered of their levels along the growth curve. Cluster 2 captured the metabolites that have their levels increased within the first hours of the growth curve and progressively decrease when growth enters the exponential phase. Most of the metabolites present in these three clusters seems to be important for the synthesis of the building blocks of the cells, as it is the case for adenine (nucleic acids); glucose, talose, mannose and trehalose (carbohydrates); succinate and hydroxypyruvate, which are precursors of TCA to generate energy and provides C skeleton for anabolic processes. Furthermore, two important nitrogen sources, asparagine and glutamine, as well as other proteogenic amino acids, such as alanine, isoleucine, proline, phenylalanine and serine were also part of these clusters. Metabolic changes related to the late stages of the growth curve were only identified in the cluster 1, including some amino acids valine, glycine, threonine, and tyrosine and other non-proteogenic amino acids like gamma aminobutyric acid (GABA), 5-oxoproline, whose levels positively correlates with the progression of the growth curve.

**Table 1:** Relative metabolite levels in the *Xanthomonas citri* cells under control used for k-means clustering.

Metabolites	Time (h)							k-means cluster
	0	1	2	4	6	12	24	
Similar to acetoacetate	1	1.0800	1.1802	1.3336	1.6184	2.2029	2.5196	1
Valine	1	1.0839	1.1636	1.3166	1.6522	2.8376	3.4291	1
Glycine	1	1.0818	1.2639	1.4811	1.9948	6.1002	15.5934	1
Threonine	1	0.9718	1.1172	1.1664	1.3529	1.6201	2.0833	1
Uracil	1	1.0293	0.9896	1.0312	0.8705	2.4626	6.4844	1
trans-4-Hydroxyproline	1	1.1968	1.2444	2.3758	4.6785	40.3410	132.5158	1
4-Aminobutanoate	1	1.2463	1.2084	1.1928	1.0709	1.1708	1.8711	1
5-Oxoproline	1	0.8994	1.0223	0.9479	1.0945	1.3349	2.1415	1
Tyrosine	1	1.0199	1.1798	1.2310	1.3618	1.9325	3.4222	1
Lactate	1	0.9160	0.9988	0.8345	0.7150	0.5465	0.5217	2
Glycerol	1	0.8150	0.8631	0.6898	0.3304	0.0320	0.0178	2
Similar to hydroxypyruvate	1	0.8982	0.9795	0.8508	0.7747	0.3372	0.2278	2
Serine	1	0.9357	1.0522	0.8838	0.7932	0.3181	0.2035	2
Asparagine	1	0.9075	1.1815	1.0515	0.9104	0.3146	0.1784	2
Shikimate	1	1.0423	0.9609	0.8819	0.6804	0.4346	0.4321	2
Adenine	1	1.0072	0.9859	0.8955	0.5836	0.0340	0.0177	2
Heptadecanoate	1	0.9402	0.9460	0.8298	0.7077	0.5165	0.4325	2
Saccharopine	1	0.8023	0.8920	0.8357	0.7209	0.2738	0.2255	2
Trehalose	1	0.7383	0.8674	0.6959	0.6531	0.2198	0.1388	2
Alanine	1	1.1296	1.1380	1.1424	1.1949	1.0470	0.6699	3
Isoleucine	1	1.0201	1.1260	1.2255	1.3914	2.1297	1.6064	3
Proline	1	1.0483	1.1667	1.2892	1.5480	3.9203	3.0405	3
Benzoate	1	0.9491	0.9801	0.8936	0.8239	0.5814	0.5164	3
Phenylalanine	1	0.9426	1.0598	1.0643	1.1590	1.6102	1.5431	3
Talose	1	0.8677	0.8738	1.1288	1.1868	1.0633	0.7141	3
Galactitol	1	0.8915	0.9160	1.0999	1.1581	1.0429	0.7371	3
Glucose	1	0.8639	0.8722	1.1220	1.1838	1.0608	0.7097	3
Glutamine	1	1.3184	1.9327	3.7215	5.6965	5.5168	2.3835	3
Spermidine	1	0.7965	0.9647	1.6143	2.2140	1.3715	1.7656	3
Similar to Glycolate	1	0.8656	0.7085	0.6009	0.4264	0.5559	0.9188	4
Succinate	1	0.3869	0.2734	0.3111	0.2009	0.1692	0.2674	4
Mannose	1	1.0389	0.9315	0.8525	0.6803	0.4217	0.4438	4

The metabolite levels of each time point were normalized to the time point 0 h (before treatment). Only metabolites which levels were significantly different from the control are displayed in this list (t-test, p-value < 0.05).

### NAC affects aminoacid metabolism in bacterial cells

**Figure 5:**
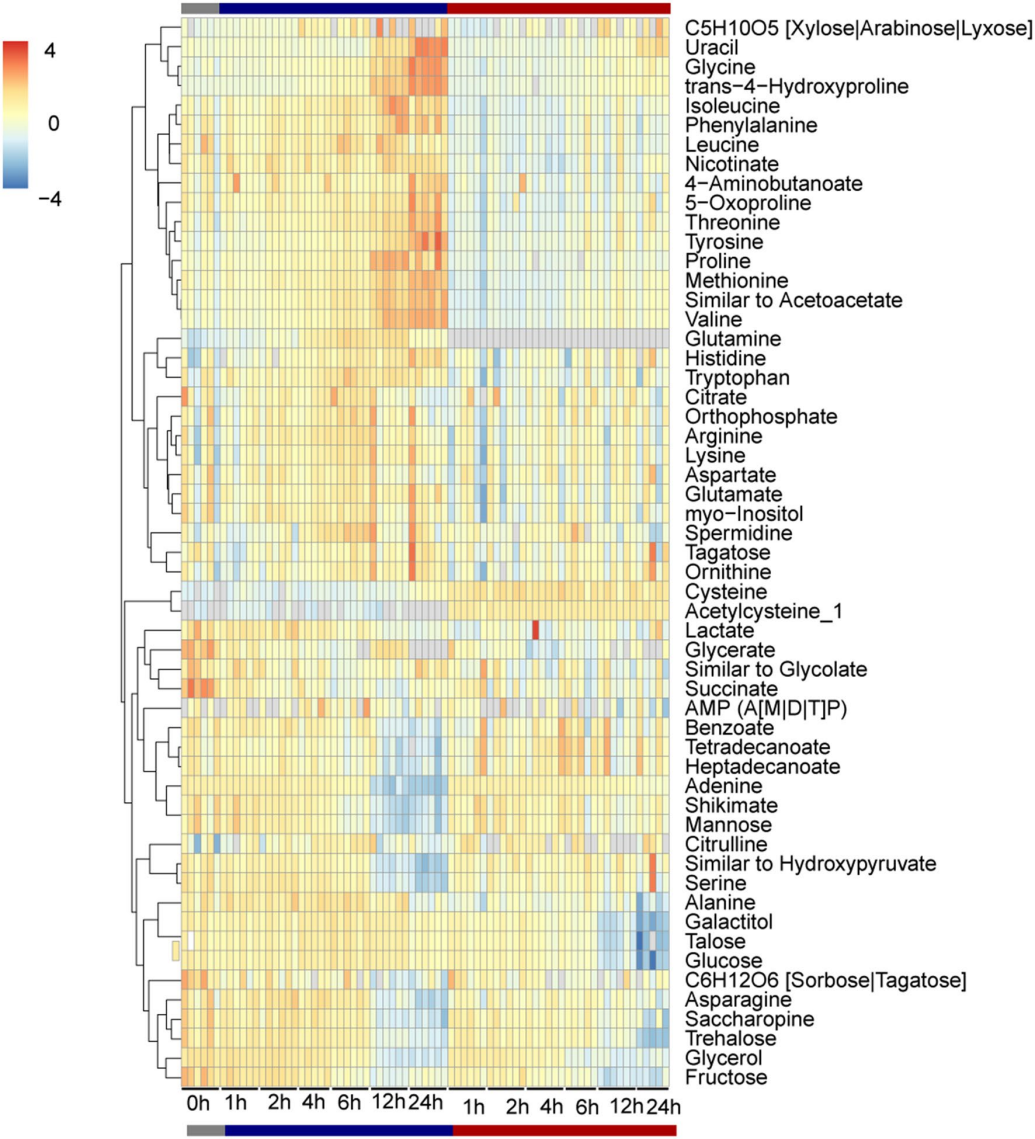
Heat map of metabolic responses to the control or NAC treated cells along 24 h *X. citri* growth. Metabolite levels were normalized by the mean of all samples from given metabolite and log_2_ transformed. Values are means of up to 5 biological replicates. Red and blue colors represent increase and decrease of metabolites, respectively. Samples are arranged according to the treatment. Metabolites are grouped according to the HCA clustering.

To dissect the possible impact of NAC on metabolism, we next compared the dynamics of the metabolites along the growth curve when cells were treated or not with this compound using a heatmap analysis (Fig. 5). As already highlighted by PCA, NAC and cysteine levels dramatically increased over time. These changes were followed by slight increases in the levels of serine and hydroxypyruvate that are precursors on the cysteine pathway. Interestingly, glutamine was completely absent in the samples treated with NAC regardless of the time point. Glutamine is an important source of nitrogen in bacteria as it is required for the synthesis of a range of nitrogen-containing compounds, including amino acids^31^.

Intriguingly, many amino acid levels (e.g., branched- and aromatic- amino acids, proline, methionine) were decreased over time in the NAC treated samples, including that NAC might interfere with nitrogen metabolism. Furthermore, cells treated with NAC seems to display a depletion in the C source in the late time points (fructose, glucose, trehalose etc.). Overall, the results suggest that the bacteria cells must metabolizing a lot of cysteine that ends up failing to eliminate so much toxicity and this affects the absorption of nutrients and the cell redox.

### Untargeted identification of biomarkers of NAC treatment

In order to discover biomarkers using both supervised model (Optimized Potentials for Liquid Simulations—OPLS) and untargeted data analysis in an integrated manner, a statistically informed molecular network approach was carried^27^. The MN allows not only to organize and classify a high number of mass spectra by similarity accelerating their annotation by comparison with spectral databases from different platforms but also allows to enrich the MN with metadata^32^.

For this, the GC-TOF/MS data processing following the workflow described in Elie, Santerre and Touboul^33^, allowed the detection of 1082 MS spectra. These were submitted to the Global Natural Products Social Network platform (GNPS) to generate a unique MN following the GC–MS workflow^34^. The spectrum of each node was searched against the spectral database available at the GNPS and allowing the annotation of 269 (25%) metabolites with cosine (similarity) score ranging from 0.98 to 0.50. Metabolites presenting cosine score > 0.75 were selected and annotated in the MN as presented below.

Subsequently, to identify untargeted markers in the MN, we integrated the statistical results from the metabolomics MVDA in the MN, when 15 features with a variables importance for the projection (VIP) value > 1.0 from the OPLS analysis (Supplementary Fig. S2) were integrated as a metadata in the MN. This metadata can be visualized though the size of the node in the MN. Such features were highlighted in the MN using larger nodes. Nodes with VIP values below 1 were kept in lower node size. In addition, the relative mass signal intensity of metabolites for NAC treatment and the untreated control samples can be visualized though the node colours. Furthermore, the relative MS signal intensity of metabolites at 0, 1, 2, 4, 6, 12 and 24 h following the NAC treatment is displayed in the bar plot next to the node. The full statistically informed MN is provided in the Supporting information (Supplementary Fig. S3).

Using this method, it was possible to identify clusters with compounds that exhibit VIP values representing variance between NAC and control groups and their dynamics over time.

**Figure 6:**
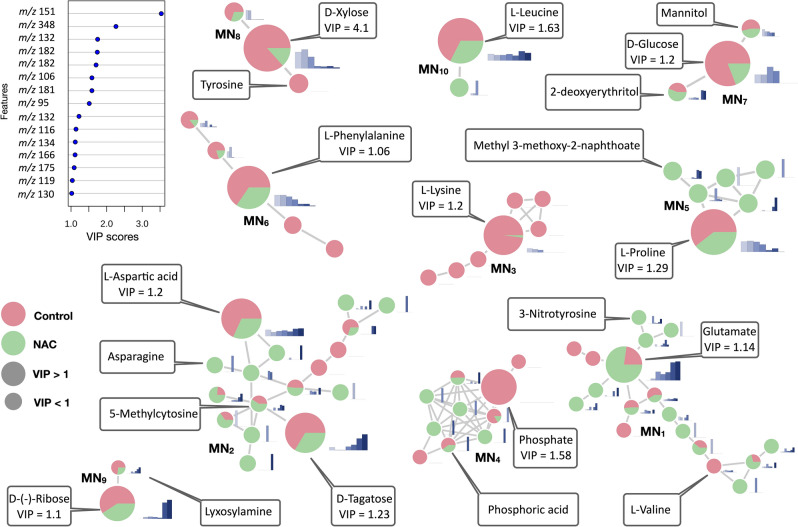
Selected clusters (MN_1_-MN_10_) from the statistically informed molecular network. VIP values greater than 1 (as seen in VIP plot) from the OPLS analysis were integrated in the molecular network and were differentiated through the node size. Larger nodes indicate features with VIP values greater than 1. Annotation of compounds was performed by comparison against Global Natural Products Social Molecular Networking spectral libraries. Green or rose indicate the relative mass signal intensity in NAC treatment and untreated control group, respectively. The bar chart next to the node in blue represents the mean relative intensity of the mass signal at times 0, 1, 2, 4, 6, 12, and 24 h of the NAC treatment group. The name of the identified metabolites with cosine > 0.75 is given in the boxes.

After analysis of the statistically informed MN, clusters were selected based on their node size. Using this criteria, 10 clusters were selected, herein named MN_1_-MN_10_. A total of 21 compounds were evidenced in the selected clusters and assigned biomarker potential. Figure 6 shows the selected clusters and annotated compounds in detail.

As demonstrated in the selected clusters, particularly to cells incubated with NAC, amino acids such as l-proline, l-leucine, l-valine, l-lysine, lyxosylamine and tyrosine were present in lower levels when compared with control. Carbohydrates as d-xylose, d-glucose, mannitol, d-tagatose, and d-ribose were present in lower levels as well and decreased over time as expected. On the other hand, metabolites such as glutamate, asparagine, methyl-3-methoxy-2-naphtoate, 5-methylcytosine and nitrotyrosine were found in high levels and to increase over time when compared to control.

These results are in agreement and complement the target approach evidencing clusters with amino acids and carbohydrates as markers for NAC treatment.

In order to map these compounds on to bacterial metabolic pathways, a list with the identified compounds in the depicted clusters from the MN was submitted to pathway impact analysis.

### Pathway impact analysis

**Figure 7:**
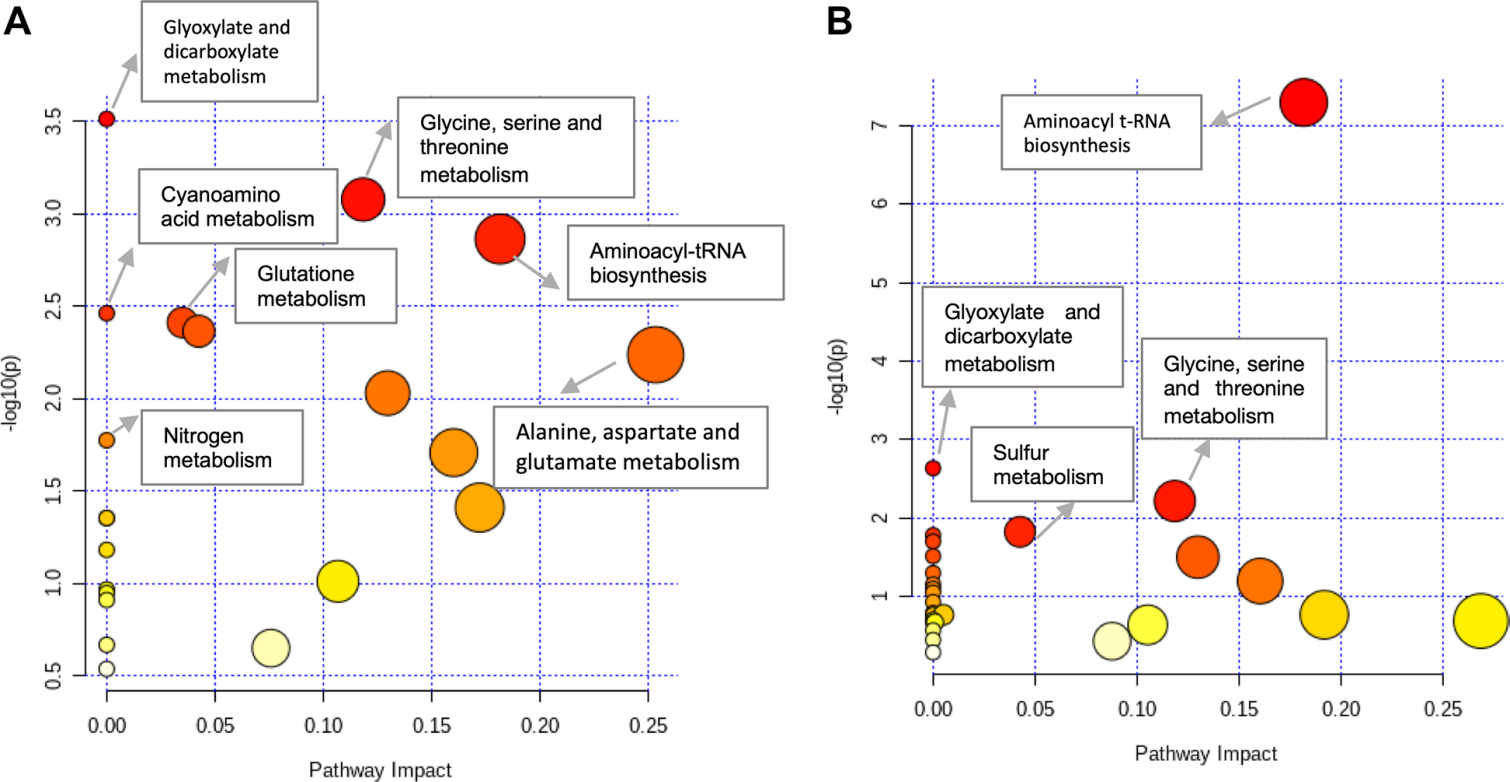
Pathway analysis of altered metabolites of *Xanthomonas citri* subsp. *citri* incubated with N-Acetylcysteine (NAC). The graph summarizes the pathways analyzed with MetaboAnalyst 4.0. (**a**) Up-regulated metabolites. (**b**) Down-regulated metabolites. Larger circles, higher and closer to Y-axes, show a higher impact of the concerned pathway of *X. citri*. Colors indicate different levels of significance. The pathways with *P* < 0.05 are presented.

In an attempt to identify deregulated pathways in which these metabolites are related, the target and untarget identified metabolites which differed significantly were mapped on to bacterial metabolic pathways. For this, a targeted analysis integrating enrichment and topology analyses was performed using the Pathway Analysis workflow available on the MetaboAnalyst (version 4.0) online platform^35^. The pathways impact analysis shows all matched pathways according to the *p* values from the pathway enrichment analysis and pathway impact values from the pathway topology analysis for up- and down-regulated metabolites separately. The identified pathways with high impact values are represented (Fig. 7). The detailed results of pathway analysis with significance levels and impact are presented in Supplementary Table S3.

This analysis showed that several pathways of amino acids, nitrogen and carbohydrate metabolism were significantly disturbed. Twelve pathways with P value < 0.05 were considered to be significantly affected after treatment with NAC. The down-regulated metabolites were located into amino sugar and nucleotide sugar metabolism and dicarboxylate metabolism, valine, leucine and isoleucine biosynthesis as well as degradation and novobiocin biosynthesis. The up-regulated metabolites were found in alanine, aspartate and glutamate metabolism, carbapenem biosynthesis, nitrogen metabolism, d-glutamine and d-glutamate metabolism, cyanoamino acid metabolism, and taurine and hypotaurine metabolism. Some of the selected metabolites which were both up- and down-regulated shared the aminoacyl-tRNA biosynthesis.

## Discussion

NAC is a cysteine analogue which has a mucolytic action due to the ability to break disulphide bridges of extracellular proteins and consequently disrupts bacterial biofilm. In addition, NAC is a well-described antioxidant and safety molecule used in medicine^16^. The molecular mechanisms underlying these two beneficial effects are well known, however, the mechanism by which NAC acts as antimicrobial is still poorly investigated. Recently it was shown that the acid pH of NAC is the key factor that facilitated the drug in entering the *Pseudomonas aeruginosa* biofilm matrix and killing the bacteria by diffusing through the cell wall. As the pH inside the bacteria is high (around 7.6), NAC dissociates and acidifies the cytoplasm, hence denaturing proteins and causing DNA damage leading to the cell death^9^. On the other hand, the authors found that there was swelling of the bacterial colonies only in the presence of NAC which confirmed that the killing of bacteria at pH 3.4 was due to the action of NAC and not merely a pH effect. Thus, to avoid the interference of acid pH, in this study we adjusted the NAC solution to pH 6.8 aiming to investigate only the antimicrobial effect of NAC in bacterial cells. The neutral pH used in our experiments could explain why it was necessary more time to kill *X. citri* (8 mg/mL of NAC for 24 h and Supplementary Fig. S1)^17^ compared with *P. aeruginosa* (10 mg/mL of NAC for 15 min)^9^ using a diluted culture to start cell growth. Likewise, no difference in the staining with SYTO9 and PI were observed in our experiments using NAC until 12 h of incubation. Thus, as PI penetrates in the disrupted cell membrane, we can conclude that the antimicrobial effect of NAC precedes cell membrane damage, since a significant percentage of cells with permeabilized membranes in presence of NAC was observed only after 24 h of incubation (Fig. 2), but the impairment in cell growth occurred just after 4 h (Fig. 1). Therefore, NAC increased the doubling time of the bacterial population, affecting the growth curve. Since there is no significative difference in the balance of potentially live and dead cells until 24 h of NAC incubation, this could result from two situations, just a small fraction of the population is actively dividing or the metabolism of the whole population of living cells is low, increasing the doubling time. In addition, our results demonstrated that NAC even at neutral pH is penetrating the bacterial cells causing alteration and decreasing cell growth (Fig. 1).

Metabolomic profiling has the potential to provide insights into the physiological drivers associated with the alterations that affect bacterial growth in presence of NAC. Indeed, our results show that the metabolome of *X. citri* cells was significantly impacted by NAC compared to control, as verified in PCA and OPLS models (Fig. 3). Taken as a whole, the NAC metabolomics signature is characterized by a decrease in amino acids such as glutamine, l-proline, l-leucine, l-valine, l-lysine, lyxosylamine and tyrosine. The amino acids l-leucine and l-valine are two of the three (l-isoleucine) essential branched-chain amino acids (BCAA) required for the growth and survival of bacteria^36^. It has been demonstrated that the enzymes belonging to the BCAA biosynthetic pathway in bacteria are an excellent potential source of targets to be explored for development of new antibacterial agents^37^. The action of NAC in BCAA could be due to its thiol group, since the threonine dehydratase/deaminase, an enzyme that plays an essential role in the biosynthetic pathway of BCAAs in microorganisms, is competitively inhibited by aminothiols^37, 38^. Therefore, the potential of NAC as an aminothiol could be better explored to interfere on BCAA biosynthesis of pathogenic bacteria. Similarly, we suggest that the decrease of the other amino acids in presence of NAC, could be also due the interference of the excess of thiol group in the enzymes involved with amino acids biosynthesis or protein synthesis. As these amino acids are sole for carbon, energy, and nitrogen resources, as well as protein syntheses, their decrease could explain the significant damage in cells growth observed in this study.

It is well known that high levels of intracellular cysteine present cytotoxicity to bacteria^39^. The increase of cysteine derived from NAC addition explains the *X. citri* growth inhibition, which could be due either to the amino acid starvation and to the oxidative DNA damage because of the cysteine cytotoxicity^40, 41^. It is worth mentioning that addition of NAC and the consequent increase of cysteine correlates with absence of glutamine and increasing of glutathione. It is known that cysteine and glutamine are both necessary to glutathione biosynthesis. Since NAC leads to an increase of cysteine levels and glutathione also increases, we suggest that the absence of glutamine results from its use for the glutathione biosynthesis, reducing its pool to a non-detectable level. This increase of cysteine due to NAC addition could change the homeostasis of intracellular cysteine, inducing oxidative stress and leading to cell death, similar to the observed by Park and Imlay^41^.

The main deregulated metabolic pathways during time course of NAC treatment were arginine and proline, alanine, aspartate and glutamate, D-glutamine and D-glutamate, purine, pyrimidine as well as nitrogen metabolisms (Fig. 5). Curiously, these pathways are somehow linked with nitrogen metabolism. In the cells inorganic nitrogen is assimilated into glutamate and glutamine, which are the major intracellular nitrogen donor^42, 43^. Glutamate is a precursor for arginine, glutamine, proline, and the polyamines. Glutamate degradation is also important for cell survival in acidic environments, and changes in glutamate concentration accompany changes in osmolarity^44^ that could cause cells death.

In our study we demonstrated that NAC without the interference of acid pH affected bacterial growth disturbing the metabolic activity, especially for carbon and nitrogen pathways. We show that NAC was able to act as antimicrobial molecule without initially affecting cell membranes but acting on targets in the cell. We suggest that, as verified for BCAA, as an aminothiol drug, NAC would be interfering with other enzymes more susceptible to competition with thiol altering its function and metabolism. In addition, the reducing property through its thiol-disulfide exchange can interact with target proteins with cysteine residue or thiol group via a thiol-disulphide exchange reaction harming protein functions^45^. In this case, NAC action is actually dependent on modulating the redox states of cysteine residues of target proteins^46^. Possibly the antimicrobial effect of NAC in bacterial cell is associate with the NAC concentration, where a dose that is high and toxic for bacteria is low and beneficial for superior organism cells^16, 18, 47^. This work shows for the first time the molecular mechanisms in which NAC works as an antimicrobial molecule. The results showed in this study open new possibilities to better explore the potential of NAC to reach specific targets improving its efficiency and specificity to kill pathogenic bacteria. It is particularly important in a scenario where the necessity of non-antibiotic drugs is increasing face the constant occurrence of resistant bacteria. In addition, NAC has a well-described antioxidant and radical scavenging activity in eukaryotic cells, it is quite stable, inexpensive, and safe^16^, features that encourage its improvement as antimicrobial compound. Even though NAC has been used for a long time in medicine, its use in agriculture was only recently investigated^8, 17, 48–50^. Thus, the potential of NAC in agriculture could be also better explored aiming to incorporate practice to control phytopathogenic bacteria or improve plant heathy to produce food in a more sustainable way^51, 52^.

## Material and methods

### Growth curve and culture conditions

Inoculation was carried out by the addition of a single isolated colony of *Xanthomonas citri* subsp. *citri* strain 306^53^ into 10 mL of Nutrient Broth Yeast extract (NBY) nutrient medium (0.5% peptone (w/v), 0.3% meat extract (w/v), 0.2% yeast extract (w/v), 0.2% K_2_HPO_4_, (w/v) and 0.05% KH_2_PO_4_ (w/v))^54^ and grown overnight at 28 °C. The cell suspension was centrifuged, and the pellet was adjusted to an optical density (OD) of 0.1 (Abs 600 nm), corresponding to a bacterial concentration of approximately 10^6^ CFU/mL. Each well of a 96-well microtiter plate containing NAC (8.0 mg/mL) was inoculated with 200 µL of the *X. citri* suspension. The growth was monitored at 0, 1, 2, 4, 6, 12, and 24 h of incubation at 28 °C by measuring the OD at 600 nm in a Varioskan Flash (Thermo Scientific). An aliquot from the same samples was collected for serial dilution and plated on NBY media. The plates were incubated at 28 °C for 48 h, followed by CFU counting. Controls (bacterial suspension without NAC) were also included in all evaluations. Three biological experiments with two technical replicates were performed.

### Live and dead

The Live/Dead Baclight kit (Thermo- Scientific L7012) was used to evaluate the bacterial membrane integrity after incubation with NAC, pH = 6.8. The experiment was performed as described by Nazaré et al.^55^. Briefly *X. citri* with an initial inoculum of 10^6^ CFU/mL were submitted to 4, 6, 12, and 24 h with 8 mg/mL of NAC, as described above. Mixtures of SYTO 9 and PI were incubated with the samples as specified (Thermo Fisher Scientific, USA). SYTO 9 (blue) can stain nucleic acids with intact or damaged membranes, while PI is an intercalating agent that stains the nucleic acid only in bacteria with damaged membranes and thus it is used to differentiate cells with permeabilized membranes. The samples were immobilized in agarose-covered slides as described by Martins et al.^56^. The cells were visualized using an Olympus BX-61 optical microscope, equipped with a monochromatic OrcaFlash- 2.8 camera (Hamamatsu, Japan). The imagens were recorded and analyzed using the software CellSens Dimension version 1.18 (https://www.olympus-lifescience.com/en/software/cellsens/). At least 1,000 cells were counted for each sample (n > 1000).

### Sample preparation

Growth of *X. citri* for metabolomic analysis in presence of 8 mg/mL of NAC, pH = 6.8 were cultured in 20 mL of NBY at 28ºC for 24 h. The growth condition was exactly the same as describe above for growth curve. Aliquots of 1 mL were collected at 0, 1, 2, 4, 6, 12, and 24 h for metabolite extraction and another aliquot was collected, plated on NBY solid medium to certify the NAC effect. For metabolite extraction, 1 mL of bacterial cell culture was sampled and filtered in polytetrafluoroethylene (PTFE) membrane with 0.22 μm pores. Bacterial pellets were extracted in 1 mL of a precooled (− 15 °C) mixture of methyl tert-butyl ether: methanol:H_2_O 3:1:1 (*v*/*v*/*v*), as previously described by Hummel et al.^57^. An aliquot of 100μL of the organic phase was dried and derivatized as described in Roessner et al.^28^.

### GC-TOF/MS analysis

An aliquot of 1 μL of each derivatized sample was injected in a splitless mode by autosampler Combi-PAL Agilent (Waldbronn, Germany) into an Agilent 7890 gas chromatograph coupled to a Pegasus II time-of-flight (TOF) mass spectrometer (Leco Corp., St. Joseph, MI, USA). Chromatogram acquisition parameters were described as Weckwerth et al.^58^. Prior to the analysis, biological samples were randomized. Quality controls, including blanks, pool of samples and mix of standards, including NAC were analyzed at the beginning of the run and after every 10 samples and were used to assess the quality of the run and performance of the equipment. Chromatograms were exported using the Leco ChromaTOF software (version 3.25) (https://www.lecosoftware.com/chromatof) in .cdf files.

### For targeted analysis

The cdf files were imported into R software^59^. Peak detection, retention time alignment, and library matching were performed using Target Search R-package^30^. Peaks were manually validated using a reference library derived from the Golm Metabolome Database^29^. Metabolites were quantified by the peak intensity of a selective mass. Metabolites intensities were normalized by dividing the OD of each biological replicate, followed by the sum of total ion count and log_2_ transformed. The data were used for HCA and PCA analyzes using the R Statistical software. The hierarchical clustering analysis was performed based on Euclidean distance and used the ggplot2 package in R Statistical Software while principal component analysis was performed using pcaMethods bioconductor package. The figures quality was improved in inkscape (version 0.92.4) (https://inkscape.org/).

### For untargeted analysis

The cdf files were then processed using MZmine 2.10 for peak detection, peak filtering, chromatogram construction, chromatogram deconvolution and alignment. The parameters used for data processing followed the method previously described by Elie, Santerre and Touboul^33^. The resulting data set (Retention time × MS signal intensities) of the 80 samples generated in a peaklist of 2235 features with associated MS spectra. This resulting peaklist was exported as input for Molecular Network multivariate data analysis and Molecular Network generation.

Pathway analysis integrating enrichment analysis was performed using global test algorithms and topology analysis using relative-betweenness centrality algorithm using *Pseudomonas putida* KT2440 as reference pathway database library.

### Molecular networking and metabolite annotation

A molecular network (MN) was created with the gas chromatography workflow available at Global Natural Products Social Molecular Networking (GNPS) (https://gnps.ucsd.edu). The spectra data was filtered by removing all MS/MS fragment ions within ± 17 Da of the precursor m/z. MS/MS spectra were window filtered by choosing only the top 6 fragment ions in the ± 50 Da window throughout the spectrum. The precursor ion mass tolerance was set to 20,000 Da and the MS/MS fragment ion tolerance to 0.5 Da. A MN was then created where edges were filtered to have a cosine score above 0.7 and more than 6 matched peaks. Further, edges between two nodes were kept in the network if and only if each of the nodes appeared in each other’s respective top 10 most similar nodes. Finally, the maximum size of a molecular family was set to 100, and the lowest scoring edges were removed from molecular families until the molecular family size was below this threshold. The library spectra were filtered in the same manner as the input data. All matches kept between network spectra and library spectra were required to have a score above 0.75 and at least 6 matched peaks. The molecular networks were visualized using Cytoscape software version 3.8.0 (https://www.cytoscape.org)^60^.

## Supplementary Information

Supplementary Table 1.

Supplementary Table 2.

Supplementary Table 3.

Supplementary Figures.
