# Supplementary material for: GC-TOF/MS-based metabolomics analysis to investigate the changes driven by N-Acetylcysteine in the plant-pathogen Xanthomonas citri subsp. citri
Source: Sci Rep. 2021 Jul 30;11:15558. doi: 10.1038/s41598-021-95113-4 (PMC8324833; doi:10.1038/s41598-021-95113-4)
Supplement: Supplementary file 3 — Supplementary Table 3. [file 41598_2021_95113_MOESM3_ESM.docx]

**Supplementary Information**

**GC-TOF/MS-based metabolomics analysis to investigate the antimicrobial activity of N-Acetylcysteine in the plant-pathogen *Xanthomonas citri* subsp. *citri***

Simone Cristina Picchi^1^, Mariana de Souza e Silva^1^, Luiz Leonardo Saldanha^2^, Henrique Ferreira^2^, Marco Aurélio Takita^1^, Camila Caldana^3*^ and Alessandra Alves de Souza^1^.

^1^Centro de Citricultura “Sylvio Moreira” – Instituto Agronômico de Campinas, Cordeirópolis, São Paulo, 13490-970, Brazil.

^2^Departamento de Bioquímica e Microbiologia, Instituto de Biociências, Universidade Estadual Paulista, Rio Claro, São Paulo, 13506-900, Brazil.

^3^ Centro Nacional de Pesquisa em Energia e Materiais (CNPEM), Campinas, São Paulo, 13083-100 Brazil

^*^Present address: Max-Planck-Institut für Molekulare Pflanzenphysiologie, Wissenschaftspark Golm, Am Mühlenberg 1, 14476 Potsdam, Germany.

**Supplementary** **Table S3.** Pathway analysis results of the altered metabolites. The analysis was performed using the MetaboAnalyst 3.0. The metabolites were analyzed separately as down- and up-regulated. The obtained *P,* -log10(p) and impact values for all pathways are presented.

|  | **Upregulated pathways** | ***P*** | **-log10(p)** | **Impact** |
| --- | --- | --- | --- | --- |
| 1 | Glyoxylate and dicarboxylate metabolism | 0.00030683 | 3.5131 | 0 |
| 2 | Glycine, serine and threonine metabolism | 0.00083592 | 3.0778 | 0.11855 |
| 3 | Aminoacyl-tRNA biosynthesis | 0.0013676 | 2.864 | 0.18182 |
| 4 | Cyanoamino acid metabolism | 0.003448 | 2.4624 | 0 |
| 5 | Glutathione metabolism | 0.0038692 | 2.4124 | 0.03498 |
| 6 | Sulfur metabolism | 0.0043135 | 2.3652 | 0.04261 |
| 7 | Alanine, aspartate and glutamate metabolism | 0.0057831 | 2.2378 | 0.25373 |
| 8 | Methane metabolism | 0.0093209 | 2.0305 | 0.12985 |
| 9 | Carbapenem biosynthesis | 0.016775 | 1.7753 | 0 |
| 10 | Cysteine and methionine metabolism | 0.019564 | 1.7085 | 0.16041 |
| 11 | D-Glutamine and D-glutamate metabolism | 0.038776 | 1.4114 | 0.17241 |
| 12 | Taurine and hypotaurine metabolism | 0.044212 | 1.3545 | 0 |
| 13 | Nitrogen metabolism | 0.044212 | 1.3545 | 0 |
| 14 | Monobactam biosynthesis | 0.0657 | 1.1824 | 0 |
| 15 | Arginine biosynthesis | 0.097175 | 1.0124 | 0.10687 |
| 16 | Pantothenate and CoA biosynthesis | 0.10747 | 0.96872 | 0 |
| 17 | Histidine metabolism | 0.11258 | 0.94854 | 0 |
| 18 | Thiamine metabolism | 0.12273 | 0.91107 | 0 |
| 19 | Pyrimidine metabolism | 0.21439 | 0.66879 | 0 |
| 20 | Arginine and proline metabolism | 0.22356 | 0.65061 | 0.07589 |
| 21 | Porphyrin and chlorophyll metabolism | 0.2895 | 0.53835 | 0 |
|  | **Downregulated pathways** | ***P*** | **-log10(p)** | **Impact** |
| 1 | Aminoacyl-tRNA biosynthesis | 5,03E-04 | 7.2988 | 0.18182 |
| 2 | Glyoxylate and dicarboxylate metabolism | 0.0023167 | 2.6351 | 0 |
| 3 | Glycine, serine and threonine metabolism | 0.0060694 | 2.2169 | 0.11855 |
| 4 | Sulfur metabolism | 0.014996 | 1.824 | 0.04261 |
| 5 | Pantothenate and CoA biosynthesis | 0.016569 | 1.7807 | 0 |
| 6 | Valine, leucine and isoleucine biosynthesis | 0.019918 | 1.7007 | 0 |
| 7 | Novobiocin biosynthesis | 0.03061 | 1.5141 | 0 |
| 8 | Methane metabolism | 0.031509 | 1.5016 | 0.12985 |
| 9 | Valine, leucine and isoleucine degradation | 0.050199 | 1.2993 | 0 |
| 10 | Cysteine and methionine metabolism | 0.063523 | 1.1971 | 0.16041 |
| 11 | D-Glutamine and D-glutamate metabolism | 0.070099 | 1.1543 | 0 |
| 12 | Nitrogen metabolism | 0.079739 | 1.0983 | 0 |
| 13 | Streptomycin biosynthesis | 0.089289 | 1.0492 | 0 |
| 14 | Monobactam biosynthesis | 0.1174 | 0.93033 | 0 |
| 15 | Cyanoamino acid metabolism | 0.16251 | 0.78913 | 0 |
| 16 | Arginine biosynthesis | 0.17127 | 0.76632 | 0 |
| 17 | Glutathione metabolism | 0.17127 | 0.76632 | 0.00514 |
| 18 | Starch and sucrose metabolism | 0.17127 | 0.76632 | 0.19185 |
| 19 | Phenylalanine metabolism | 0.19707 | 0.70538 | 0 |
| 20 | Alanine, aspartate and glutamate metabolism | 0.2055 | 0.68718 | 0.26866 |
| 21 | Thiamine metabolism | 0.21386 | 0.66987 | 0 |
| 22 | Phenylalanine, tyrosine and tryptophan biosynthesis | 0.21386 | 0.66987 | 0.00092 |
| 23 | Tyrosine metabolism | 0.23033 | 0.63765 | 0.10526 |
| 24 | Pentose phosphate pathway | 0.27015 | 0.5684 | 0 |
| 25 | Pyrimidine metabolism | 0.35818 | 0.4459 | 0 |
| 26 | Arginine and proline metabolism | 0.37187 | 0.42961 | 0.0878 |
| 27 | Purine metabolism | 0.51674 | 0.28673 | 0 |
