# Supplementary material for: GC-TOF/MS-based metabolomics analysis to investigate the changes driven by N-Acetylcysteine in the plant-pathogen Xanthomonas citri subsp. citri
Source: Sci Rep. 2021 Jul 30;11:15558. doi: 10.1038/s41598-021-95113-4 (PMC8324833; doi:10.1038/s41598-021-95113-4)
Supplement: Supplementary file 4 — Supplementary Figures. [file 41598_2021_95113_MOESM4_ESM.pdf]

Supplementary Information

GC-TOF/MS-based metabolomics analysis to investigate the antimicrobial activity of N-Acetylcysteine in the plant-pathogen *Xanthomonas citri* subsp. *citri*

Simone Cristina Picchi[1], Mariana de Souza e Silva[1], Luiz Leonardo Saldanha[2], Henrique Ferreira[2], Marco Aurélio Takita[1], Camila Caldana[3*] and Alessandra Alves de Souza[1].

[1]Centro de Citricultura "Sylvio Moreira" – Instituto Agronômico de Campinas, Cordeirópolis, São Paulo, 13490-970, Brazil.
[2]Departamento de Bioquímica e Microbiologia, Instituto de Biociências, Universidade Estadual Paulista, Rio Claro, São Paulo, 13506-900, Brazil.
[3] Centro Nacional de Pesquisa em Energia e Materiais (CNPEM), Campinas, São Paulo, 13083-100 Brazil
[*]Present address: Max-Planck-Institut für Molekulare Pflanzenphysiologie, Wissenschaftspark Golm, Am Mühlenberg 1, 14476 Potsdam, Germany.

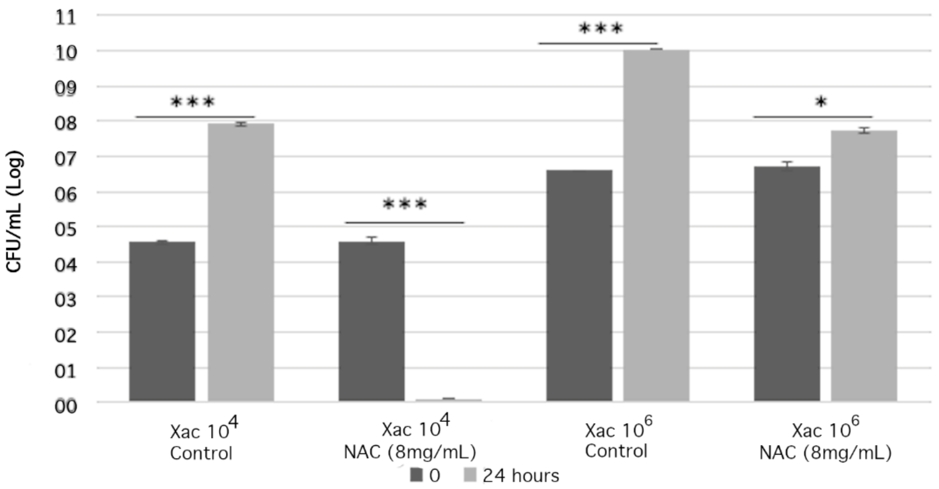

Figure S1. Effect of *N*-acetylcysteine (NAC) on the viability of *Xanthomonas citri* upon different initial cell concentrations. Bacterial cells were grown for 24 h without NAC (Control) or in the 8 mg/mL of NAC. The data are the means of three independent

experiments. The bars represent standard deviations of the means. Asterisks indicate significant differences with *$P < 0.05$ or *** $P < 0.001$ (*t*-test) compared to the control.

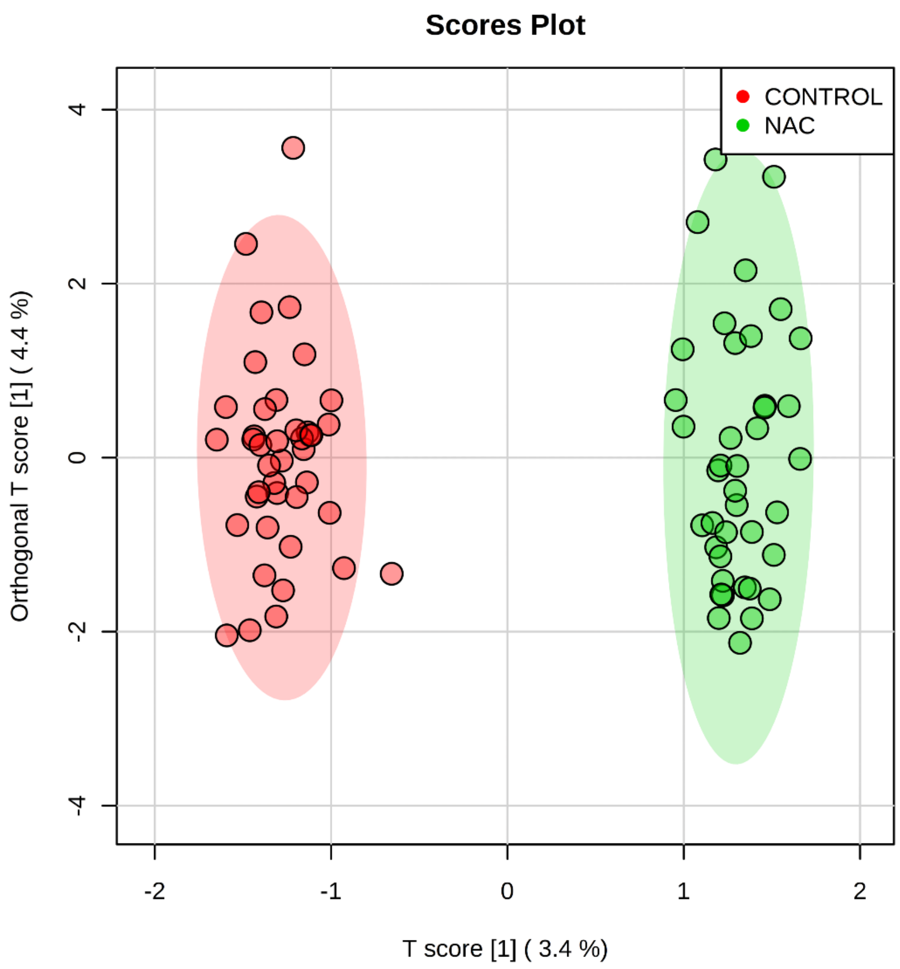

Figure S2. Multivariate analysis of the GC-TOF/MS data. OPLS score plot showing the control samples (red) NAC-treated samples (green).

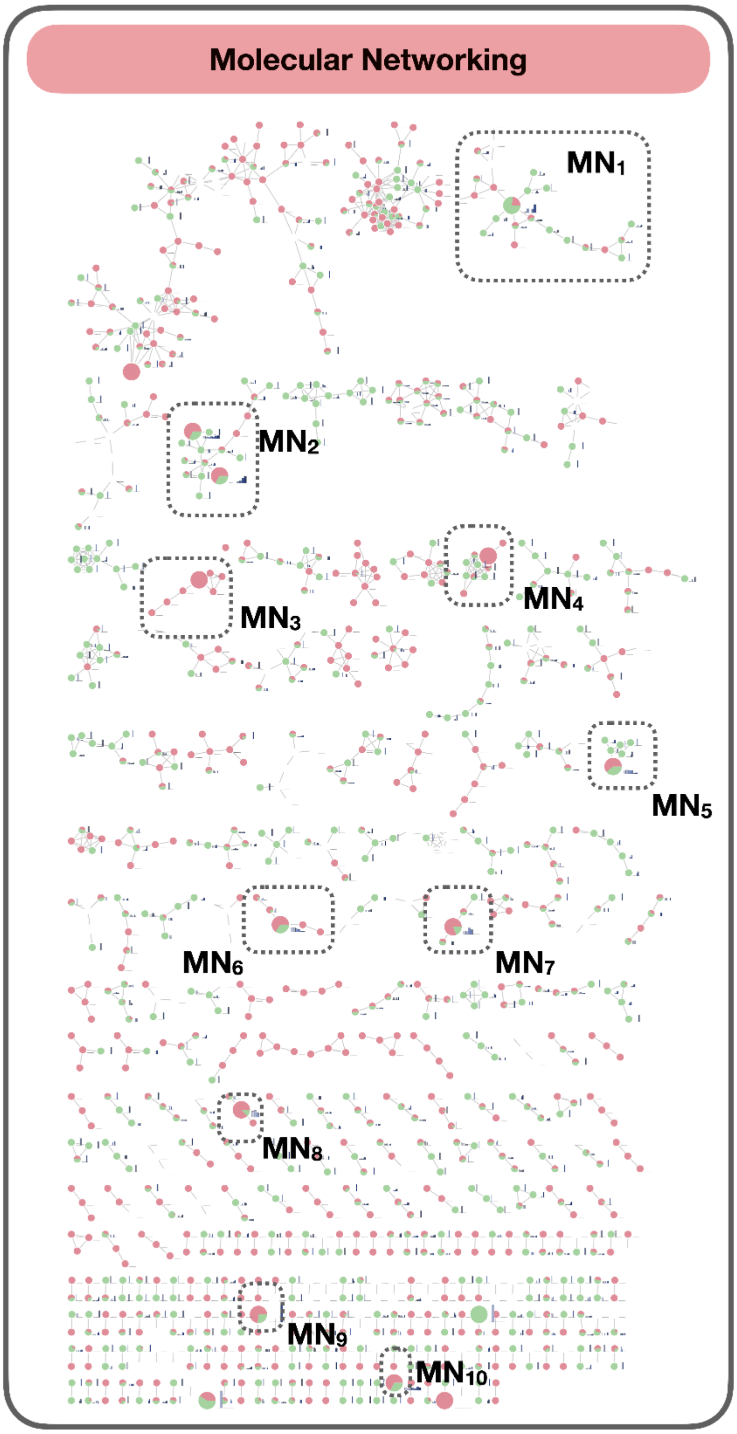

Figure S3. *Statistically-informed molecular networking* generated by integrating the MVDA results to the MN. The VIP values from the OPLS analysis were integrated in the MN and can be visualized through the size of the node. Nodes with higher size indicate features with VIP values > 1. Clusters (MN_1-MN_{10}) were selected based on their node size and are highlighted in dotted line boxes.
